# Correlation of exhaled propofol with Narcotrend index and calculated propofol plasma levels in children undergoing surgery under total intravenous anesthesia - an observational study

**DOI:** 10.1186/s12871-021-01368-9

**Published:** 2021-05-26

**Authors:** Sebastian Heiderich, Tara Ghasemi, Nils Dennhardt, Robert Sümpelmann, Vanessa Rigterink, Katja Nickel, Oliver Keil, Dietmar Böthig, Christiane E. Beck

**Affiliations:** 1 Clinic of Anesthesiology and Intensive Care Medicine, Hannover Medical School, Carl-Neuberg-Str. 1, 30625 Hannover, Germany; 2 Department for Pediatric Cardiology and Intensive Care, Hannover Medical School, Hannover, Germany

**Keywords:** Exhaled propofol, Children, TIVA, Pediatric anesthesia, Pharmacokinetic modeling

## Abstract

**Background:**

Exhaled propofol concentrations correlate with propofol concentrations in adult human blood and the brain tissue of rats, as well as with electroencephalography (EEG) based indices of anesthetic depth. The pharmacokinetics of propofol are however different in children compared to adults. The value of exhaled propofol measurements in pediatric anesthesia has not yet been investigated. Breathing system filters and breathing circuits can also interfere with the measurements. In this study, we investigated correlations between exhaled propofol (exP) concentrations and the Narkotrend Index (NI) as well as calculated propofol plasma concentrations.

**Methods:**

A multi-capillary-column (MCC) combined with ion mobility spectrometry (IMS) was used to determine exP. Optimal positioning of breathing system filters (near-patient or patient-distant) and sample line (proximal or distal to filter) were investigated. Measurements were taken during induction (I), maintenance (M) and emergence (E) of children under total intravenous anesthesia (TIVA). Correlations between ExP concentrations and NI and predicted plasma propofol concentrations (using pediatric pharmacokinetic models Kataria and Paedfusor) were assessed using Pearson correlation and regression analysis.

**Results:**

Near-patient positioning of breathing system filters led to continuously rising exP values when exP was measured proximal to the filters, and lower concentrations when exP was measured distal to the filters. The breathing system filters were therefore subsequently attached between the breathing system tubes and the inspiratory and expiratory limbs of the anesthetic machine. ExP concentrations significantly correlated with NI and propofol concentrations predicted by pharmacokinetic models during induction and maintenance of anesthesia. During emergence, exP significantly correlated with predicted propofol concentrations, but not with NI.

**Conclusion:**

In this study, we demonstrated that exP correlates with calculated propofol concentrations and NI during induction and maintenance in pediatric patients. However, the correlations are highly variable and there are substantial obstacles: Without patient proximal placement of filters, the breathing circuit tubing must be changed after each patient, and furthermore, during ventilation, a considerable additional loss of heat and moisture can occur. Adhesion of propofol to plastic parts (endotracheal tube, breathing circle) may especially be problematic during emergence.

**Trial Registration:**

The study was registered in the German registry of clinical studies (DRKS-ID: DRKS00015795).

## Introduction

Total intravenous anesthesia (TIVA) has over the years been demonstrated to have many advantages in pediatric anesthesia, with decreased postoperative emergence agitation compared to volatile anesthetics, and a major reduction in postoperative nausea and vomiting [1, 2]. In particular, TIVA has also been very beneficial for sedation and short interventional procedures with spontaneous breathing. Because of the physiological maturation process during childhood, the characterization of pharmacokinetics and pharmacodynamics of propofol dosing in the pediatric population is still challenging, and clinical feedback is necessary. Electroencephalography (EEG) based monitoring can provide cerebral pharmacodynamic feedback of the hypnotic effect, but drug concentrations may only be estimated from pharmacokinetic models. There is still a lack of real-time measurements of anesthetic concentrations of propofol in blood.

Exhaled propofol (exP) measurements may reflect a real-time estimation of plasma concentration and can be useful in the clinical setting. It has been demonstrated that there is a good correlation between EEG-based anesthesia depth and exP concentrations in adults [3]. In addition, exP concentrations correlate well with propofol concentrations in adult human blood [4–6]. Moreover, in an animal model, they correlate well with concentrations in the central nervous system [7]. However, all experiments in literature were carried out with adult patients. In this study, we investigated the correlation between exP and EEG-based monitoring as well as propofol plasma concentrations calculated using pediatric pharmacokinetic models in pediatric anesthesia.

## Methods

This prospective observational study was conducted according to the standards set forth by the Declaration of Helsinki and Good Clinical Practice guidelines. It was approved by the local ethics committee (Ethics Committee of Hannover Medical School, Germany, Chairperson Prof. Dr. S. Engeli, No. 7955_BO_K_2018 on July 25th, 2018), registered in the German registry of clinical studies (DRKS-ID: DRKS00015795), and conducted between November 2019 and July 2020 at the Clinic for Anaesthesiology and Intensive Care Medicine of the Hannover Medical School, Germany.

### Multi-capillary-column ion mobility spectrometry

A multi-capillary-column (MCC) combined with ion mobility spectrometry (IMS) (Edmon® Exhaled Drug Monitor, B.Braun Melsungen AG, Melsungen, Germany) was used to determine the exP. The operating gas for the MCC-IMS was systematically processed from ambient air by the Redmon® air purifier (B.Braun Melsungen AG, Melsungen Germany) [8]. The MCC-IMS covers a range between 1 and 20 ppb of exP with an accuracy of ±10% at 20 ppb [9]. In this study datapoints were recorded at 5-min intervals.

### Impact of breathing system filters

**Fig. 1:**
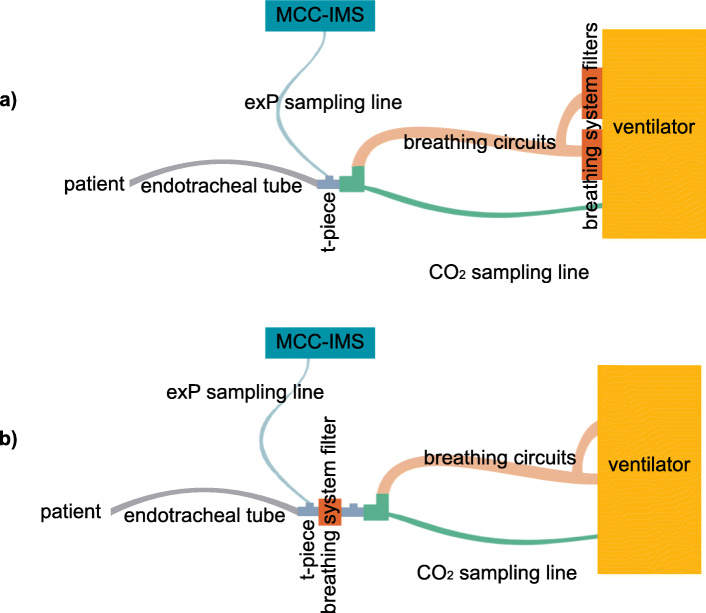
Experimental setup: **a**) Main experiment: breathing filters were positioned directly at the inspiratory and expiratory limbs of the ventilator (patient-distant positioning). **b**) Preliminary breathing filter test: the breathing system filter was positioned at the tube (near-patient positioning). MCC-IMS: Multi-capillary-column ion mobility spectrometer, exP: exhaled propofol

For the prevention of infections and preservation of heat and moisture during anesthesia, the use of breathing system filters with a > 99% retention for airborne particles, and liquids retention to at least 60 hPA or 20 hPA above the selected maximum ventilation pressure in the anesthetic system is recommended [10]. These principles allow using breathing circuits multiple times over up to 7 days. In pediatric anesthesia, filters of various sizes are used, depending on the child’s weight. In this preliminary experiment, we studied the impact of three different sizes of filters (type 1: Humid-Vent Mini® (heat and moisture exchange, 0–6 kg of body weight); type 2: Humid-Vent Filter Pedi® (bacterial, viral heat and moisture exchange, 7–35 kg of body weight), Teleflex Medical, Athlone, Ireland; type 3: Ultipor® breathing system filter (bacterial, viral, heat and moisture exchange, > 35 kg of body weight), Pall Medical, Fribourg, Switzerland. The filters were first positioned at their normal position between airway device and breathing circuit (Fig. 1b). During a steady state (20 min of unchanged propofol infusion rate), the changes in exP concentration were measured. Then, filters were removed and sampling continued. Because filter size type 2 is used most frequently in pediatric anesthesia, the measurement was also carried out before and behind the filter, all during a steady-state situation.

### Exhaled propofol measurement

The study population as per the inclusion criteria comprised stable children with American Society of Anesthesiologists (ASA) physical status I-III scheduled for elective surgery under endotracheal intubation and total intravenous anesthesia. Written informed consent was obtained from the parents. If not already in place, intravenous access was established outside the operating room (OR). Propofol bolus injection (T0) was performed with 1 mg·kg^− 1^ outside the OR and repeated until the children were comfortable with being separated from their parents. Simultaneously, propofol infusion was started with 6 mg·kg^− 1^·h^− 1^, keeping the children comfortable under light sedation. After positioning on the operating table, the standard monitoring consisting of electrocardiography, non-invasive blood pressure measurement and pulse oximetry was established (Carescape B850, GE Healthcare, General Electric Company, Boston, USA). Thereafter, EEG monitoring was established before induction of anesthesia with impedances below 6000 Ohm. The EEGs of each patient were recorded continuously with the EEG monitor Narcotrend Compact M® (software version 3.3; MT MonitorTechnik, Bad Bramstedt, Germany). After preoxygenation, anesthesia was induced with another bolus injection of propofol adapted by the attending anesthesiologist, and a remifentanil infusion (0.3 μg·kg^− 1^·min^− 1^) as well as a single dose of atracurium (0.5 mg·kg^− 1^) were administered. After endotracheal intubation, the ventilator was adjusted to the children’s age and weight to maintain normocapnia (end-tidal CO_2_ = 35–40 mmHg), and the measurement of the exP concentration was started. A continuous sampling flow (200 ml·min^− 1^) was taken from a T-piece between endotracheal tube and breathing circuit.

A polyethylene-free sampling tube then connected the T-piece to the MCC-IMS. Once a minute, the MCC-IMS measured propofol concentrations of the sample flow stream. The breathing system filters were positioned directly on the ventilator to avoid interference with the measurement. A breathing circuit suitable for infants, children and adults was used (Double D Circuit, 180 mm, Teleflex, Dublin, Ireland).

Fresh gas flow of the anesthetic workstation was therefore adjusted 0.5 l·min^− 1^ higher than the patients’ minute ventilation to avoid rebreathing of propofol. Breathing filters (Pall Breathing System Filter, Pall Medical, Fribourg, Switzerland) were positioned directly onto the connectors of the ventilator (Primus®, Drägerwerk AG & Co. KG, Lübeck, Germany) to avoid an accumulation or backlog of propofol, as our preliminary testing had shown that filters represent a disturbance factor of unknown range. The experimental setup is illustrated in Fig. 1a.

Propofol and remifentanil infusion rates were adjusted by the attending anesthesiologist in order to maintain anesthesia according to the Narcotrend index (NI) (recommended range D0-E2) and the usual clinical signs (movements, change in heart rate and blood pressure).

A warm air blanket was used to maintain normothermia during the procedure (Moeck Warming System®, Moeck, Hamburg, Germany). All standard monitoring parameters, NI, ventilation parameters, temperature, propofol and remifentanil infusion and exhaled propofol concentration were documented at predefined points in time: start of propofol (T0) followed by five-minute intervals thereafter for 1 h (induction and maintenance of anesthesia), and again for the last 20 min of anesthesia until extubation (emergence). All events, such as intubation, propofol bolus injection and change of propofol infusion, were recorded separately. For maintenance infusion, 10 ml·kg^− 1^·h^− 1^ of a balanced isotonic electrolyte solution with 1% glucose (E 148 G1 Päd, Serumwerk Bernburg, Bernburg, Germany) was administered. The exP measurements were interrupted after 60 min if the operation and extubation could not be completed within 80 min. For those cases, an additional breathing system filter suitable for the child’s weight and tidal volume was connected between tube and breathing circuit, and low-flow ventilation was established to avoid ongoing loss of heat and moisture. If possible (stable propofol infusion for at least 30 min with assumed steady state), the additional type-2 filters were removed prior to emergence, and measurements were resumed until extubation.

### Data analysis

All recorded data were analyzed using MS Excel (Excel 2010; Microsoft, Seattle, USA), GraphPad Prism (Prism 7; Graph Pad Software Inc., San Diego, USA) and MedCalc (MedCalc Statistical Software version 17.4, MedCalc Software bvba, Ostend, Belgium) software tools. According to a post hoc power analysis, a sample size of 26 patients would result in a 95% power to detect the described correlation between exP, calculated plasma concentration of propofol and NI, with an error probability of 5%.

Normal distribution was checked with the D’Agostino-Pearson’s test. Demographic and procedural data were presented as median [interquartile range] or frequency (percentage). Pearson correlation and regression analysis were performed with a predefined significant level of α = 0.05. Weight, height, age and propofol infusion data of enrolled patients were used to calculate simulated propofol plasma concentrations with a TIVA simulation program (TIVA Trainer, version 9.1, 2014) [11], using the Paedfusor [12, 13] and Kataria [14] data sets.

## Results

### Position variations of breathing system filters

**Fig. 2:**
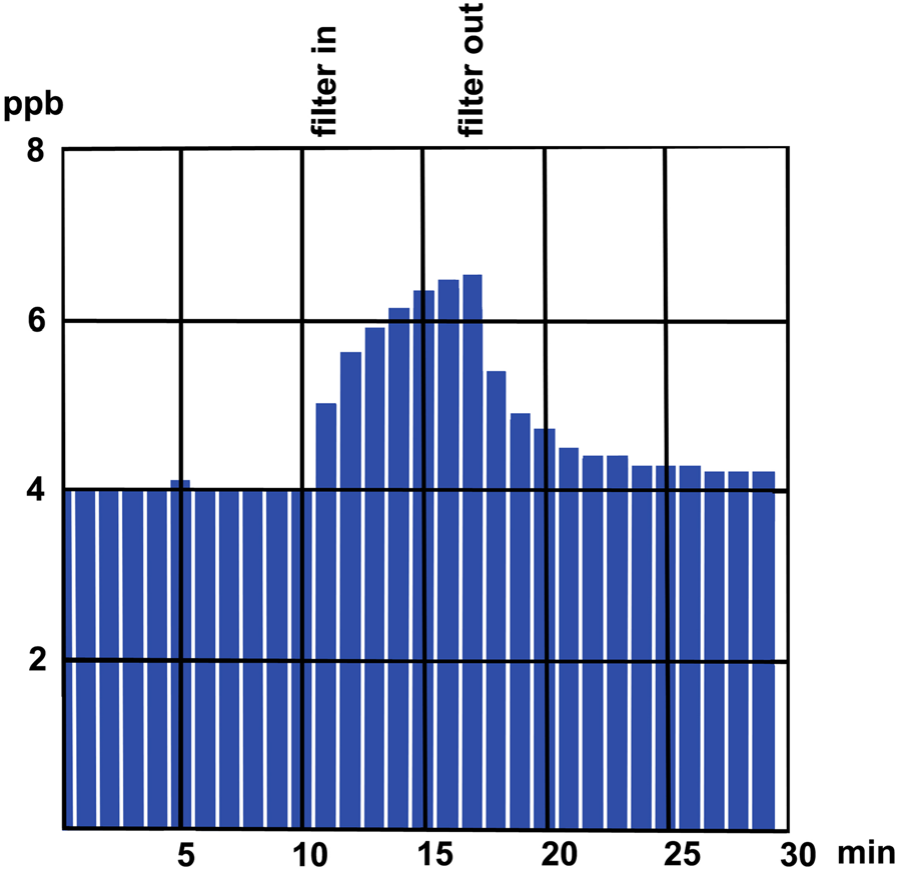
Example of increase and decrease of exhaled propofol concentration after inserting and removing a type-2 filter during stable propofol infusion

Inserting a type-1 filter into the breathing circuit resulted in a maximum drop in exP of 0.4 ppb, while inserting type-2 and 3 filters led to a mean increase in the measured exP of 2.3 (0.9–3.8) ppb within 5 min, with a further increase over 30 min, which slowed down, but did not reach a steady state. After removing type-2 and 3 filters, exP concentrations decreased within 5 min by a mean 1.95 (1.1–4.1) ppb to almost the former steady-state values (this course is exemplified in Fig. 2). Simultaneous measurements of exP concentrations in a steady-state situation before and behind type-2 filters showed a difference of 5.5 ppb (65.5%), with the lower values measured behind the filter.

### Exhaled propofol measurement

**Fig. 3:**
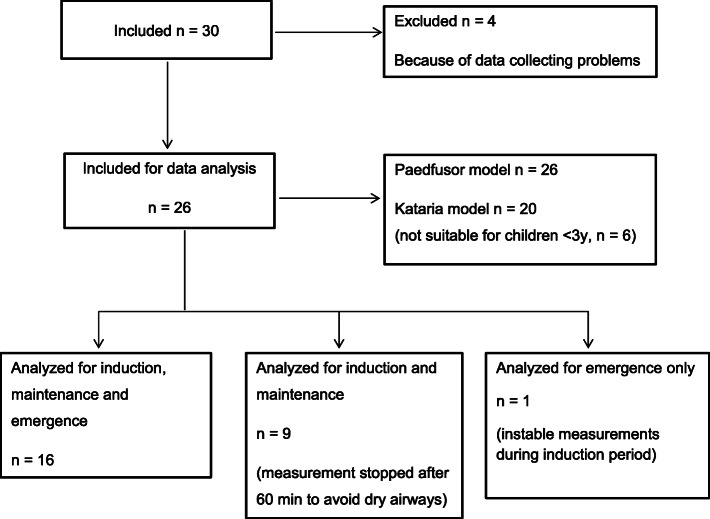
Study flow chart

Data sets of 30 children were analyzed in this study. Four children had to be excluded due to incomplete data collection. The data sets of 16 children were analyzed for the induction (I), maintenance (M) and emergence (E) of anesthesia. Nine cases were analyzed for I and M; for these cases, the emergence phase was excluded from analysis because the surgery took longer than 60 min, and the airways therefore had to be prevented from drying out due to high-flow ventilation and patient-distant filtering. For 1 case, only the emergence (E) phase was analyzed. A detailed study flow chart is shown in Fig. 3. Patient characteristics and procedural data are listed in Table 1. The Kataria model is not approved for children under 3 years of age (*n* = 6). For these cases, only the Paedfusor model was used. For all other cases, both models have been applied.

**Table 1:** Demographic data: sex, ASA status, age, weight, and surgical procedures are presented as number or median [IQR]. ASA: American Society of Anesthesiologists physical status classification system; M: male; F: female; y: years, NI: Narcotrend index

Patients	
Sex (M/F)	10/16
Age (y)	4 (0–8)
Weight (kg)	18 (6–45)
ASA I/II/III	6/16/4
Surgical procedures	
Abdominal	11
Vascular	8
Urological	6
Thoraxic	1
NI	45 [33–55] (15–82)
Endtidal pCO_2_	36 [34–39]

Reliable exP values were available approximately 5 min after intubation of the patients; for that reason, complete data sets for all patients were available 15 min after propofol infusion was started outside the OR. The median continuous propofol infusion rate was 10 mg·kg^− 1^·h^− 1^ (range 4–14), the median NI was 45 IQR [33–55], with 90.6% of the values in the range for recommended depth of anesthesia between D0 and E2.

**Fig. 4:**
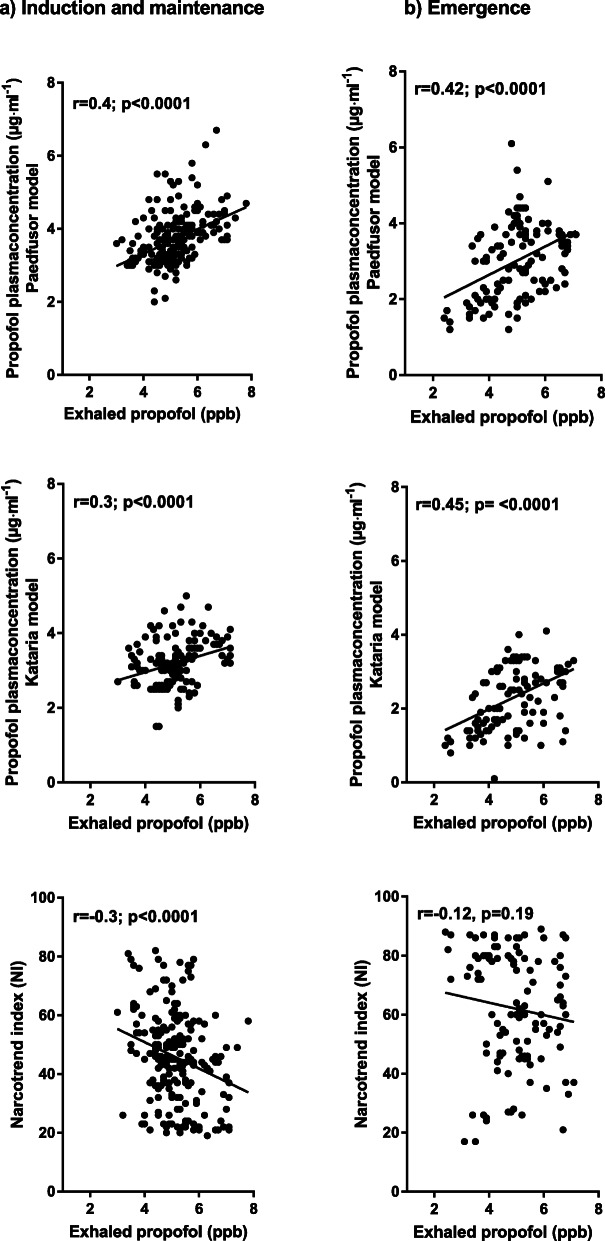
Scatter plots of exhaled propofol concentration in parts per billion (ppb) and propofol plasma concentration (μg·ml^− 1^) calculated by the Paedfusor model and Kataria model for induction (I) and maintenance (M) of anesthesia and emergence (E) from anesthesia (r = correlation coefficient)

For the IM period, the Pearson correlation between exP and NI (*n* = 221) was r = − 0.3, 95% CI − 0.4 to − 0.1; *P* < 0.0001; between exP and the plasma concentration calculated using the Paedfusor model (n = 221), the Pearson correlation was r = 0.4, 95% CI 0.3 to 0.5; *P* < 0.0001; and for exP and the plasma concentration calculated using the Kataria model (*n* = 153), the Pearson correlation was r = 0.3, 95% CI 0.2 to 0.5; *P* < 0.0001 (Fig. 4). For the time period encompassing E from anesthesia, including the last 20 min before extubation, the Pearson correlation between exP and NI (*n* = 122) was r = − 0.1, 95% CI − 0.3 to 0.1; *P* = 0.3; between exP and the plasma concentration calculated using the Paedfusor model (n = 122), r = 0.42, 95% CI 0.3 to 0.6; *P* < 0.0001; and between exP and the plasma concentration calculated using the Kataria model, r = 0.45, 95% CI 0.3 to 0.6; *P* < 0.0001 (*n* = 108) (Fig. 4).

**Fig. 5:**
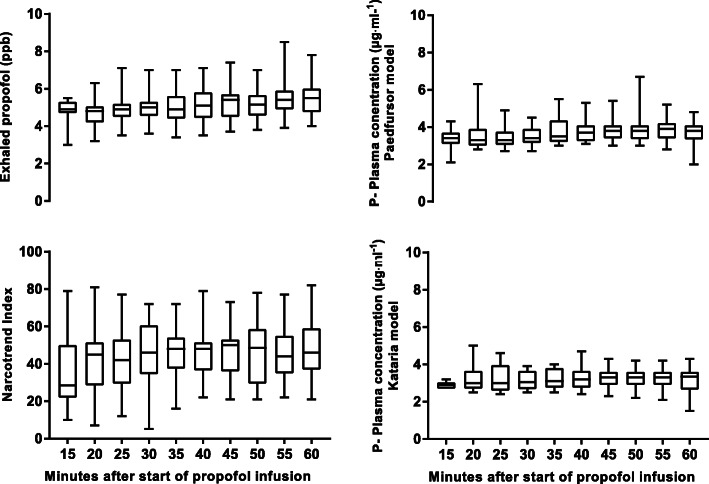
Box plots of exhaled propofol, Narcotrend Index and propofol (P) plasma concentration calculated by the Paedfusor and Kataria model during induction and maintenance. The data are expressed as median values and 25/50 percentiles. The whiskers represent the highest and the lowest value

Comparing the course of anesthesia over time, the median NI at IM was 45, and it increased to 63 at E (difference between medians 17, 95% CI 14 to 23, *P* < 0.0001). The median calculated plasma concentration of propofol decreased from 3.5 to 3.0 μg·ml^− 1^ when using the Paedfusor model (difference between medians − 0.4, 95% CI − 0.9 to − 0.5; *P* < 0.0001) and from 3.2 to 2.4 μg·ml^− 1^ when using the Kataria model (difference between medians − 0.8, 95% CI − 1.1 to − 0.6, *P* < 0.0001). The median concentration of exP was 5.1 ppb at IM and 5.0 ppb at E, *P* = 0.13. Figure 5 shows the range and quartiles of exP, NI and calculated plasma concentrations using both models over the course of I and M.

## Discussion

In this study, we demonstrated that, in hemodynamically stable children without impaired lung function, exP concentrations correlate significantly with NI during induction and maintenance. It also showed a significant correlation with calculated propofol blood concentrations during induction, maintenance and emergence of pediatric anesthesia.

For the wider pediatric population, TIVA requires calculation and validation of pharmacokinetic models adapted to the pediatric population. These models need to integrate the physiological maturation during childhood, which influences pharmacokinetics and metabolism, and the interindividual variability in the pediatric population using covariants such as age, weight and height [15].

The Paedfusor model is a prototype of a target infusion system developed in 1998, and was evaluated in 29 children aged 1 to 15 years [12, 13]. The Kataria model is one of the most commonly used models in children between 3 and 11 years [14]. In this study, we demonstrated that exP concentrations correlate with calculated propofol plasma levels using both the Kataria and the Paedfusor models. The correlation, however, is lower than that of blood levels in adults and animal models [4–7]. The ethical hurdles for clinical studies on children are too high, and intraoperative blood sampling for study purposes requires a sound justification. As a rule, healthy and stable children are not equipped with an arterial catheter, which would be available for intraoperative blood sampling, unless the operation is likely to be associated with hemodynamic instability due to a higher amount of volume displacement or for other reasons. For that reason, we had to compare exP levels with assumed plasma concentrations, calculated using the Kataria and Paedfusor models. However, the exP concentration may reflect not solely the blood and tissue concentration, but also be affected by physiological breathing parameters. Dead space ventilation, minute ventilation and fresh gas flow can influence the exP concentration. This may limit its accuracy in some clinical settings [16]. Still, we do not believe that this had a relevant impact in our study: The lower correlation in this study compared to adult studies may be explained by the fact that we compared exP with calculated propofol concentrations using pharmacokinetic models. This also reflects the main limitation of this study: future experiments may reveal the correlation to true propofol blood concentrations.

The correlation between NI and exP is also lower in this study than in studies on adults, where it was about r = 0.7 when compared to Bispectral index (BIS) monitoring [17, 18]. It is known from studies in children that children < 2 years display higher NI and greater interindividual variability than children > 2 years [19]. Nevertheless, the Narcotrend is highly reliable in showing the depth of sedation in children [20]. This provides a comparison of exP with two parameters that reliably represent the depth of anesthesia. The results of this study demonstrate that the interindividual variation of exP is lower than the interindividual variation of NI in children (Fig. 5). Therefore, it may be a superior tool to avoid awareness, but it could also mean that variations in blood and effect-site concentrations are less likely to be picked up in the exhaled setting. Therefore, the relationship between plasma concentration, brain tissue concentration and exP concentration requires further evaluation and pharmacokinetic modeling for the time delay between changes in plasma concentration and effects on tissue concentration or exP concentration [21, 22]. Because volatile propofol binds and adsorbs reversibly to various types of plastic with saturation kinetics, the measurement itself remains a great challenge in the anesthesia setting, with its different plastics in face masks, endotracheal tubes, laryngeal masks and breathing circuits. Lorenz at al. demonstrated the behavior of propofol in breathing circuits in an ICU setting where propofol was outgassed from the breathing circuit for hours and remained at 2.8 ppb after 60 h of washing out [23]. In laboratory studies, perfluoroalkoxy alkanes (PFA) have been identified as materials with low adsorption of propofol [24]. Therefore, real-time exP measurements may be optimized by the use of PFA for airway devices and breathing circuits. This study demonstrated another problem: the use of near-patient breathing system filters leads to an immediate increase in exP when measured between patient and filter. We assume that volatile propofol is either reflected or adsorbed by the breathing system filter itself because concentrations behind the filter remained low. This cofounder can be avoided by attaching the filters directly to the expiratory and inspiratory limb of the anesthetic workstation [9]. Unfortunately, this practice requires changing the breathing circuit after each patient, which is cost-expensive and time-consuming.

## Conclusions

Our study shows that exP correlates with calculated propofol concentrations (using Kataria and Paedfusor models) and NI during induction and maintenance of total intravenous anesthesia in pediatric patients. The results of the exP measurements were influenced by the type and position of different standard breathing filters. Still, some obstacles still need to be addressed: the cost of changing breathing circuits after each patient, and loss of heat and moisture due to patient-distant breathing system filters; adsorption and release of propofol in standard breathing circuits and airway devices. Furthermore, correlation of exP to real propofol plasma levels in children needs to be evaluated. Once these obstacles have been overcome, randomized controlled studies should aim to evaluate whether real-time measurements of exP help to avoid awareness and shorten emergence from anesthesia.

## Data Availability

The datasets used and/or analyzed during the current study are available from the corresponding author on reasonable request.

## Abbreviations

ASA: American society of anesthesiologists
BIS: Bispectral index
E: Emerging from anesthesia
EEG: Electroencephalography
exP: Exhaled propofol
hPA: Hectopascal
ICU: Intensive care unit
I: Induction of anesthesia
IMS: Ion mobility spectrometry
M: Maintenance of anesthesia
MCC: Multi-capillary-column
NI: Narcotrend index
OR: Operating room
PPB: Parts per billion
PFA: Perfluoroalkoxy alkane(s)
T0: Start of propofol injection
TIVA: Total intravenous anesthesia

## References

[1] Chandler JR, Myers D, Mehta D, Whyte E, Groberman MK, Montgomery CJ, Ansermino JM (2013). Emergence delirium in children: a randomized trial to compare total intravenous anesthesia with propofol and remifentanil to inhalational sevoflurane anesthesia. Paediatr Anaesth.

[2] Cohen IT, Finkel JC, Hannallah RS, Hummer KA, Patel KM (2003). Rapid emergence does not explain agitation following sevoflurane anaesthesia in infants and children: a comparison with propofol. Paediatr Anaesth.

[3] Colin P, Eleveld DJ, van den Berg JP, Vereecke HEM, Struys M, Schelling G, Apfel CC, Hornuss C (2016). Propofol breath monitoring as a potential tool to improve the prediction of intraoperative plasma concentrations. Clin Pharmacokinet.

[4] Takita A, Masui K, Kazama T (2007). On-line monitoring of end-tidal propofol concentration in anesthetized patients. Anesthesiology.

[5] Perl T, Carstens E, Hirn A, Quintel M, Vautz W, Nolte J, Jünger M (2009). Determination of serum propofol concentrations by breath analysis using ion mobility spectrometry. Br J Anaesth.

[6] Hornuss C, Praun S, Villinger J, Dornauer A, Moehnle P, Dolch M, Weninger E, Chouker A, Feil C, Briegel J, Thiel M, Schelling G (2007). Real-time monitoring of propofol in expired air in humans undergoing total intravenous anesthesia. Anesthesiology.

[7] Müller-Wirtz LM, Maurer F, Brausch T, Kiefer D, Floss M, Doneit J, et al. Exhaled Propofol concentrations correlate with plasma and brain tissue concentrations in rats. Anesth Analg. 2020. 10.1213/ANE.0000000000004701 32118620

[8] B Braun Melsungen AG M, Germany: REDMON instructions for use. Air Purifier for Exhaled Drug Monitor EDMON. 2019;005E:1-32.

[9] B Braun Melsungen AG M, Germany: EDMON operating instructions for operating theatre and intensive care unit. 2018;107E:1-52.

[10] Kramer A, Kranabetter R, Rathgeber J, Züchner K, Assadian O, Daeschlein G, et al. Infection prevention during anaesthesia ventilation by the use of breathing system filters (BSF): Joint recommendation by German Society of Hospital Hygiene (DGKH) and German Society for Anaesthesiology and Intensive Care (DGAI). GMS Krankenhaushyg Interdiszip. 2010;5(2):Doc13. 10.3205/dgkh000156 PMC2951096 20941333

[11] Engbers F SN, Kenny CNC: TIVA Trainer (Copyright) v. 9.1. 2014.

[12] Absalom A, Kenny G (2005). 'Paedfusor' pharmacokinetic data set. Br J Anaesth.

[13] Absalom A, Amutike D, Lal A, White M, Kenny GN (2003). Accuracy of the 'Paedfusor' in children undergoing cardiac surgery or catheterization. Br J Anaesth.

[14] Kataria BK, Ved SA, Nicodemus HF, Hoy GR, Lea D, Dubois MY, Mandema JW, Shafer SL (1994). The pharmacokinetics of propofol in children using three different data analysis approaches. Anesthesiology.

[15] Constant I, Rigouzzo A (2010). Which model for propofol TCI in children. Paediatr Anaesth.

[16] Wirtz LM, Kreuer S, Volk T (2019). Hüppe T: [modern breath analysis]. Med Klin Intensivmed Notfmed.

[17] Liu Y, Gong Y, Wang C, Wang X, Zhou Q, Wang D, Guo L, Pi X, Zhang X, Luo S (2015). Online breath analysis of propofol during anesthesia: clinical application of membrane inlet-ion mobility spectrometry. Acta Anaesthesiol Scand.

[18] Buchinger H, Kreuer S, Hellbrück R, Wolf A, Fink T, Volk T, Bödeker B, Maddula S, Baumbach JI (2013). Minimal retarded Propofol signals in human breath using ion mobility spectrometry. Int J for Ion Mobil Spectrom.

[19] Dennhardt N, Boethig D, Beck C, Heiderich S, Boehne M, Leffler A, Schultz B, Sumpelmann R (2017). Optimization of initial propofol bolus dose for EEG Narcotrend index-guided transition from sevoflurane induction to intravenous anesthesia in children. Paediatr Anaesth.

[20] Davidson AJ (2007). Monitoring the anaesthetic depth in children – an update. Curr Opin Anesthesiol.

[21] Kreuer S, Hauschild A, Fink T, Baumbach JI, Maddula S, Volk T (2014). Two different approaches for pharmacokinetic modeling of exhaled drug concentrations. Sci Rep.

[22] Sahinovic MM, Struys M, Absalom AR (2018). Clinical pharmacokinetics and pharmacodynamics of Propofol. Clin Pharmacokinet.

[23] Lorenz D, Maurer F, Trautner K, Fink T, Hüppe T, Sessler DI, Baumbach JI, Volk T, Kreuer S (2017). Adhesion of volatile propofol to breathing circuit tubing. J Breath Res.

[24] Maurer F, Lorenz DJ, Pielsticker G, Volk T, Sessler DI, Baumbach JI, Kreuer S (2017). Adherence of volatile propofol to various types of plastic tubing. J Breath Res.

